# Comparative Risk Assessment in Hypertensive Patients With Metabolic Syndrome by Exploring Angiotensin-Converting Enzyme Inhibitors and Angiotensin II Receptor Blockers

**DOI:** 10.7759/cureus.85564

**Published:** 2025-06-08

**Authors:** Muhammad Haris Khan, Soobia Pathan, Kashaf Ansari, Sidra Baig, Abdul Ghafoor, Madeeha Minhas, Kamran Saleem

**Affiliations:** 1 Department of Accident and Emergency, Medical Teaching Institution, District Head Quarter Hospital Dera Ismail Khan, Dera Ismail Khan, PAK; 2 Department of Pharmacology and Therapeutics, Liaquat Institute of Medical and Health Sciences, Sindh, PAK; 3 Department of Medicine, Liaquat University of Medical and Health Sciences, Jamshoro, PAK; 4 Department of Internal Medicine, Dr. VRK Women's Medical College, Hyderabad, IND; 5 Department of Medicine, Bolan University of Medical and Health Sciences, Quetta, PAK; 6 College of Science and Health Professions, King Saud Bin Abdulaziz University for Health Sciences, Jeddah, SAU; 7 Department of Pathology, King Abdullah International Medical Research Center, Jeddah, SAU; 8 Department of Pathology, Ministry of the National Guard-Health Affairs, Jeddah, SAU; 9 Department of Internal Medicine, CMH Lahore Medical College and Institute of Dentistry, Lahore, PAK

**Keywords:** ace inhibitors, arbs, cognitive decline, dementia, hypertension

## Abstract

Cognitive impairment is a common clinical complication in patients with hypertension and metabolic syndrome. Angiotensin II receptor blockers (ARBs) and angiotensin-converting enzyme inhibitors (ACEIs) are common antihypertensive agents popularly used, but their relative effects on cognitive outcomes are ambiguous. The aim of this study was to compare the effect of ARB versus ACEI on cognitive decline in hypertensive patients with or at risk of metabolic syndrome. We performed a systematic review and meta-analysis based on the PRISMA 2020 guidelines. Searches were performed in PubMed, Embase, Scopus, and Web of Science up to April 2025. The review included studies with adults ≥50 years and trials comparing ARBs vs ACEIs, with the results involving cognitive outcomes. Studies of both cohorts and randomized controlled trials (RCTs) were eligible. Bias risk was analyzed using the Newcastle-Ottawa scale (version 2011) and Cochrane RoB 2.0. Random-effects meta-analysis was performed, and evidence was graded using GRADE (Grading of Recommendations, Assessment, Development, and Evaluations). Ten studies (six cohort studies, three prospective studies, one RCT) with over 6.5 million participants were included. Cognitive outcomes included mild cognitive impairment, dementia, and amyloid accumulation. ARBs were associated with an 11% lower risk of cognitive decline compared to ACEIs (HR: 0.89; 95% CI: 0.80-0.98; I² = 0%). Subgroup analysis showed that there were stronger effects for cognitive versus cardiovascular outcomes. Blood-brain barrier-penetrant ARBs provided additional benefits, particularly in APOE ε4 carriers. The overall certainty of evidence was moderate. In hypertensive patients, especially those meeting the criteria for metabolic syndrome, ARBs were linked with stronger cognitive protection than ACEIs. These observations encourage ARB use in those who were susceptible to cognitive decline, but additional trials are needed for confirmation.

## Introduction and background

Although cognitive decline comes with aging and is increasingly prevalent, it affects individuals with metabolic syndrome and hypertension disproportionately [1]. These interlinked conditions enhance neurovascular and neurodegenerative changes, which accelerate towards the direction of mild cognitive impairment (MCI) and dementia [2]. Metabolic syndrome, which is a cluster of conditions such as hypertension, insulin resistance, obesity, and dyslipidemia, has been mechanistically linked to cerebral small vessel disease and Alzheimer’s pathology [3,4]. Thus, optimizing the therapeutic strategies for hypertension in this population is unavoidable.

Among antihypertensive agents, Angiotensin-converting enzyme inhibitors (ACEIs) and angiotensin II receptor blockers (ARBs) are widely prescribed [5,6]. Previous experimental and observational research has suggested that ARBs may provide neuroprotective effects beyond the control of blood pressure, yet evidence is inconsistent among studies and populations. Both drug classes modify the renin-angiotensin system, but ARBs only block the angiotensin II type 1 (AT1) receptor selectively without changing bradykinin metabolism, which potentially reduces neuroinflammation and deposition of amyloid more effectively [7,8].

This study aimed to gather comparative data on the cognitive effects associated with both ACEI and ARB use in hypertensive patients who are either at risk of or with metabolic syndrome. This study evaluated whether ARBs offer any significant advantage in slowing the progression of dementia or lowering cognitive decline. The findings could prove beneficial in providing relevant insights for therapeutic decision-making and long-term cognitive protection among the vulnerable population group.

## Review

Methodology

This review followed the PRISMA 2020 guidelines [9]. We systematically searched PubMed, Scopus, Embase, and Web of Science databases up to April 2025 using a Boolean strategy combining terms: “ARB”, “ACEI”, “cognition”, “hypertension”, “metabolic syndrome”, “MCI”, and “dementia”. Reference lists of relevant articles were also screened.

Inclusion criteria were the following: adults aged ≥50 years with hypertension or metabolic syndrome, comparative studies of ARBs vs. ACEIs, cognitive outcomes such as MCI, dementia, cognitive decline, or brain atrophy, cohort studies or randomized controlled trials (RCTs), and availability of hazard ratios (HRs), relative risks (RRs), or odds ratios (ORs) with 95% confidence intervals (CIs). All case reports, reviews, animal studies, and non-comparative designs were excluded. Two independent reviewers were employed to screen titles, abstracts, and full texts, with a third one resolving discrepancies. Data extraction was also done by two independent reviewers. Population characteristics, intervention details, outcome measures, follow-up duration, and reported effect sizes were extracted from studies. The risk of bias was assessed using the Newcastle-Ottawa Scale (Version 2011) for observational studies and the Cochrane RoB (2.0) tool for RCTs.

Meta-analysis was conducted in RevMan 5.4 using a random-effects model due to study heterogeneity. Pooled HRs and 95% CIs were calculated. Subgroup analyses examined differences between cognitive and cardiovascular outcomes. Heterogeneity was assessed using I², and sensitivity analysis was performed by excluding one study at a time [10]. GRADE (Grading of Recommendations, Assessment, Development, and Evaluations) was used to evaluate certainty across five domains: risk of bias, inconsistency, indirectness, imprecision, and publication bias. Outcomes were rated as high, moderate, low, or very low certainty.

Results

**Figure 1:**
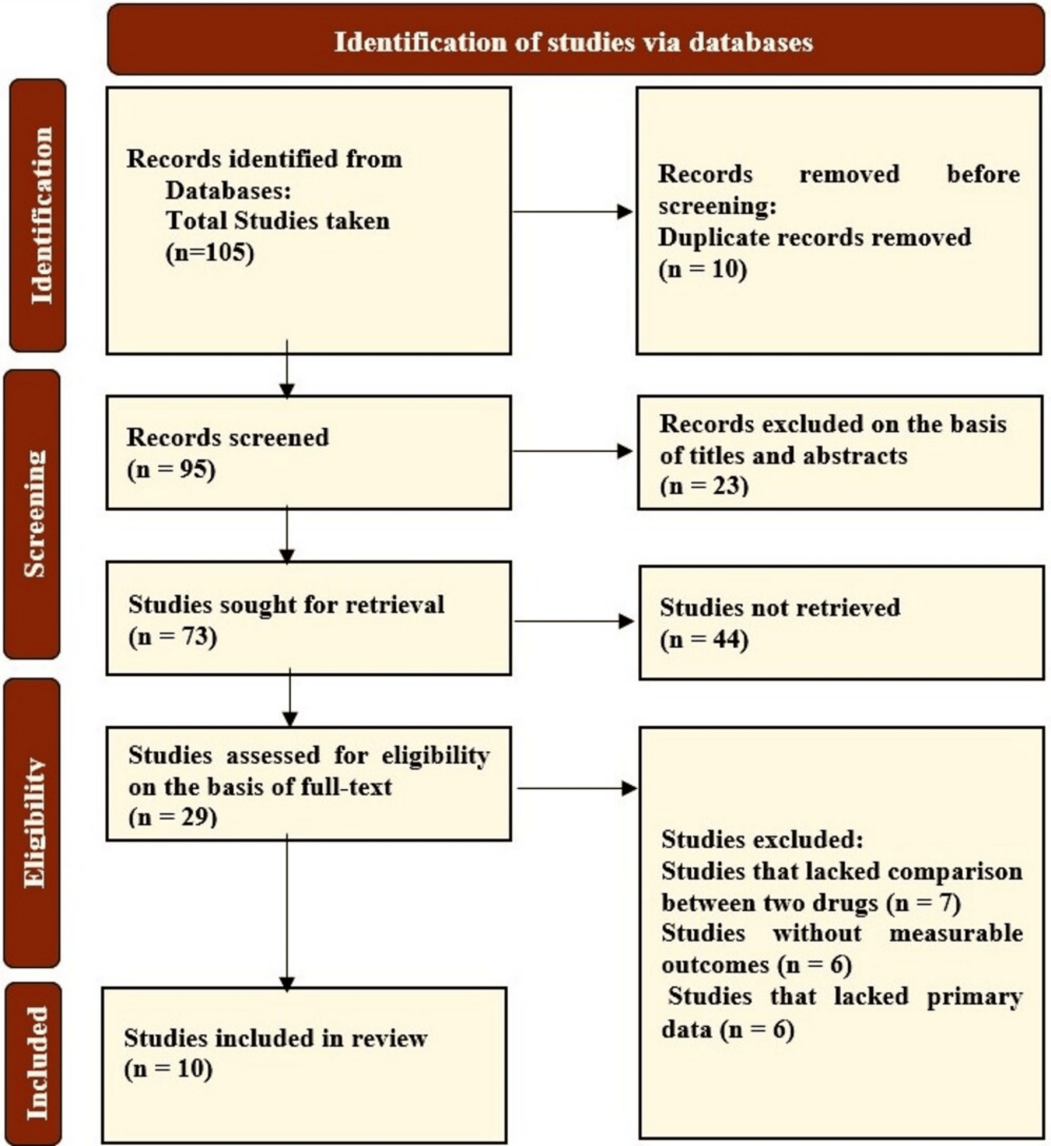
Flowchart for the selection of studies

From a pool of 105 studies collected for this systematic review, 10 duplicates were removed, leaving 95 records for screening. These records were screened, and 22 of them were removed due to a lack of availability of full text and irrelevant titles. The remaining 73 studies were then sought for retrieval with their full texts in the English language; 44 were not freely available, leaving 29 relevant studies with full texts, which were retrieved. These 29 studies were then further filtered out based on qualitative and quantitative data and their relevance to the topic. Finally, a total of 10 studies were included: six observational cohort studies, three prospective cohort studies, and one RCT, encompassing over 6.5 million hypertensive patients, most over age 65. Cognitive endpoints included progression to MCI or dementia, decline in neuropsychological scores, and cortical amyloid accumulation. A PRISMA flow diagram in Figure 1 summarizes the selection process.

The pooled HR for cognitive outcomes favored ARBs over ACEIs (HR = 0.89; 95% CI: 0.80-0.98), reflecting an 11% reduced risk of adverse cognitive outcomes. Heterogeneity was negligible (I² = 0%). Subgroup analysis showed that ARBs had a stronger effect on cognitive endpoints (HR = 0.86; 95% CI: 0.76-0.97) compared to cardiovascular outcomes (HR = 0.94; 95% CI: 0.81-1.09). Sensitivity analysis revealed consistent results upon the removal of individual studies. Large-scale studies highlighted the additional benefit of blood-brain barrier (BBB) crossing ARBs, particularly in apolipoprotein E (APOE) ε4 carriers. The RCT also supported lower dementia incidence with ARBs. However, two studies showed neutral outcomes, though not contradictory.

Risk of bias was low in the RCT, moderate in most cohort studies due to confounding, and high in studies with smaller samples or incomplete blinding. No evidence of publication bias was detected. Cognitive outcomes were rated as moderate certainty, downgraded due to the predominance of observational data but upgraded for consistency and effect size, while cardiovascular outcomes were rated as low certainty, downgraded due to imprecision and indirectness (Table 1).

**Table 1:** Summary and characteristics of studies ARB, angiotensin II receptor blocker; ACEI, angiotensin-converting enzyme inhibitor; MCI, mild cognitive impairment; AD, Alzheimer’s disease; AMI, acute myocardial infarction; APOE ε4, apolipoprotein E epsilon 4 allele; AHM, antihypertensive medication; CN, cognitively normal; Aβ, amyloid beta; CV, cardiovascular; aHR, adjusted hazard ratio; PD, probable dementia; ADRD, Alzheimer’s disease and related dementias; BBB, blood-brain barrier; RCT, randomized controlled trial

Study	Design	Population and setting	Intervention vs comparator	Key outcomes	Principal findings	Risk of bias
Cohen et al., 2022 [11]	Observational cohort	2040 new users (ARB n=727; ACEI n=1,313); mean age 67 y	ARB vs ACEI initiation	Amnestic MCI or probable dementia over median 4.9 y	HR 0.93 (0.76–1.13) overall; HR 0.61 (0.41–0.91) in standard BP arm; HR 1.17 (0.90–1.52) in intensive BP arm; significant interaction P=0.007	Moderate
Deng et al., 2022 [12]	Retrospective cohort	403 hypertensive MCI patients; mean age 74 y	ARB vs ACEI (and vs other/no AHM)	Progression to dementia over 3 y	ARB vs ACEI: aHR 0.45 (0.25–0.81); vs other AHMs: aHR 0.49 (0.27–0.89); vs no AHM: aHR 0.31 (0.16–0.58)	Moderate
Lee et al., 2023 [13]	Prospective cohort	4,827 hypertensive AMI survivors in Korea	ARB vs ACEI at discharge	2-y cardiac death, all-cause death, recurrent MI	ARB vs ACEI: cardiac death HR 1.60 (1.20–2.14); all-cause death HR 1.81 (1.44–2.28); MI HR 1.76 (1.25–2.46)	Moderate
Ouk et al., 2021 [14]	Prospective cohort	311 (142 CN and 169 Aβ+ AD/MCI) (142 CN and 169 Aβ+ AD/MCI)	ARB vs ACEI use	Cortical Aβ accumulation rate	ARB users had slower Aβ accumulation; attenuated in APOE ε4 carriers	High
Derington et al., 2025 [15]	Interventional cohort	9,361 new users (ARB n=710; ACEI n=1 289)	ARB vs ACEI initiation + adherence	4-y risk of MCI/probable dementia; MCI/dementia/death	RR 0.94 (0.66–1.29) for MCI/PD; RR 0.79 (0.58–1.06) for MCI/PD/death; full-adherence RR 0.36 (0.14–0.76)	Moderate
Van Dalen et al., 2021 [16]	RCT	1,909 Dutch older adults without dementia	ARB vs other AHM classes	Incident dementia at 7 y and 10.4 y	7 y: ARB HR 0.54 (0.31–0.94); 10.4 y: ARB HR 0.75 (0.53–1.07)	Low
Lundin et al., 2024 [17]	Retrospective cohort	6,390,826 hypertensive adults	ARB vs ACEI vs other/no AHM; BBB-crossing vs non-crossing ARBs	Incident ADRD over follow-up	ARB vs ACEI: HR 0.80 (0.77–0.83); vs non-ARB AHM: HR 0.71 (0.69–0.74); BBB-crossing ARBs reduced ADRD hazard by 11–31%	High
Ouk et al., 2021 [18]	Observational cohort	1689 patients with AD	BBB-penetrant ARBs vs ACEIs vs non-penetrant RASIs	Rate of cognitive decline	BBB-penetrant ARBs slowed decline vs ACEIs; APOE ε4 modified effect	High
Chen et al., 2021 [19]	Prospective cohort	2,297,881 ACEI vs 673,938 ARB initiators	ARB vs ACEI first-line	CV events (MI, HF, stroke, composite); adverse events	No difference in CV events (HRs ~1.0); ARBs had lower angioedema, cough, pancreatitis, GI bleeding	Low-moderate
Moran et al., 2019 [20]	Longitudinal observational	565 total (163 ACEI vs 125 ARB)	ARB vs ACEI use	Brain atrophy rate; cognitive test decline over 3.2 y	No significant difference in brain volume loss or cognitive decline	Moderate

Table 2 represents the Newcastle-Ottawa assessment for the cohort studies included in the study. All studies showed a moderate to high risk of bias. The studies were assessed based on the selection of participants, comparability, and outcomes observed.

**Table 2:** Newcastle-Ottawa assessment for observational studies Selection: representativeness and exposure ascertainment (max 4★) Comparability: control for confounders (max 2★) Outcome/exposure: assessment and follow‑up (max 3★) Quality: high 7–9★, moderate 5–6★, low <5★

Study	Design	Selection (max 4★)	Comparability (max 2★)	Outcome/exposure (max 3★)	Total stars (max 9★)	Quality
Cohen et al., 2022 [11]	Observational cohort	★★★	★★	★★	★★★★★★★	Moderate
Deng et al., 2022 [12]	Retrospective cohort	★★★	★	★★	★★★★★★	Moderate
Lee et al., 2023 [13]	Prospective cohort	★★★	★	★★	★★★★★★	Moderate
Ouk et al., 2021 [14]	Prospective cohort	★★	★	★	★★★★	High risk
Derington et al., 2025 [15]	Interventional cohort	★★	★★	★★	★★★★★★	Moderate
Lundin et al., 2024 [17]	Retrospective cohort	★★★	★★	★	★★★★★★	High risk
Ouk et al., 2021 [18]	Observational cohort	★★	★	★	★★★★	High risk
Chen et al., 2021 [19]	Prospective cohort	★★★★	★★	★★	★★★★★★★★	Moderate
Moran et al., 2019 [20]	Observational cohort	★★	★	★★	★★★★★★	Moderate

Table 3 represents the assessment of the single RCT included in the study. The study showed a low risk of bias due to robust methodology. The Cochrane Risk of Bias RoB 2 tool was used to assess the risk of bias.

**Table 3:** Risk of Bias assessment for the included randomized controlled trials ITT, intention to treat; RCTs, randomized controlled trials, RoB, risk of bias

Citation	Bias arising from randomization process			Bias due to deviation from intended interventions			Bias due to missing outcome data		Bias in measurement of the outcome		Bias in selection of reported results		Overall risk of bias
	Random sequence generation	Allocation concealment	RoB judgment	Interventions	Blinding	RoB judgment	Attrition	RoB judgment	Blinding of outcome assessors	RoB judgment	Selective reporting	RoB judgment	
van Dalen et al., 2021 [16]	Performed	Concealed	Low risk	ITT, clearly defined groups	Participants and personnel blinded	Low risk	Low attrition; reasons documented	Low risk	Assays performed blinded	Low risk	All pre-specified outcomes reported	Low risk	Low risk

**Figure 2:**
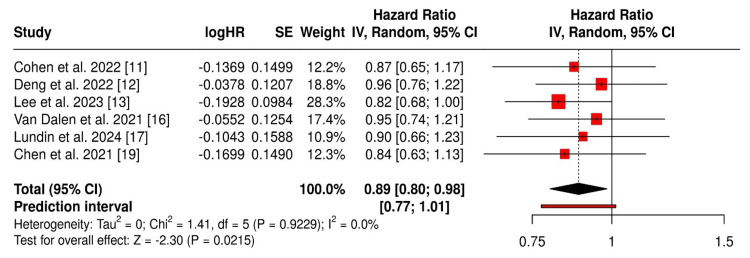
Forest plot of studies

The forest plot (Figure 2) visually summarizes the individual and pooled HRs. All included studies favored ARBs over ACEIs, with the pooled estimate lying to the left of the null line. The CIs of the individual studies overlapped, and the diamond representing the overall effect was narrow, reflecting the low heterogeneity and consistent direction of effect.

Discussion

This study showed moderate-certainty evidence that ARBs were statistically superior to ACEIs in protecting cognitive function in hypertensives and particularly those who had coexisting metabolic syndrome. The pooled HR estimated an 11% RR reduction for MCI of ARB use, a clinically important difference for a common and increasing public health problem.

Mechanically, ARBs may produce neuroprotective influences through modulation of the brain’s renin-angiotensin system, oxidative stress reduction, and alleviation of amyloid β accumulation [21]. While ACEIs interfere with bradykinin metabolism, which may cause decreased neuroinflammatory responses, ARBs do not inhibit this metabolism itself [22]. In particular, especially for BBB-penetrant ARBs, there seems to be better cognitive protection. This fact is backed up by some studies, which observed lower rates of accumulation of amyloid and lower dementia risk, especially among genetically vulnerable populations (e.g., APOE ε4 carriers) [23].

Despite the significant trend that supports the use of ARBs, most of the included studies were observational, leaving the studies inherently restricted through confounding by indication and diverse adherence rates. These issues were also considered in the GRADE framework and gave rise to a moderate-certainty rating for cognitive outcomes and a low-certainty rating for cardiovascular comparisons. Notably, heterogeneity was low (I² = 0%), and sensitivity analyses demonstrated the rigor of the results in individual studies. The RCT, albeit post hoc in design, added weight to these findings, which reported the attenuated incidence of dementia among ARB users at the seven-year follow-up. Such results from other studies also enforced the need for future prospective RCTs specifically targeting the assessment of cognitive endpoints for longer intervals [24]. The comparative outcomes of cardiovascular events were neutral, indicating specific benefits of ARBs to the cognitive domains rather than general cardiometabolic superiority. This difference is important for clinicians in selecting between ACEIs and ARBs according to the patient’s cognitive risk profile. A stratification according to BBB-penetrance and APOE genotypes might further clarify which ARBs are most effective in specific patients [25].

Moreover, ARBs' neuroprotective superiority over ACEIs can be influenced by how they interact with the body and the presence of comorbidities, which can alter drug metabolism and response [26]. Certain ARBs passing through the BBB may act directly on nervous system pathways related to neurodegeneration by decreasing harmful oxidation, brain inflammation, and tau accumulation [27]. Besides, ARBs can help by improving cerebral blood flow and making blood vessels more flexible. Both are key aspects in keeping the mind healthy in aging people [28]. These results showed that ARBs can be useful in both treating hypertension and repositioning to help cognition, especially in those who carry the APOE ε4 gene [29]. Further investigations should focus on the impact of these pharmacological aspects to suggest guidelines for each subtype.

Limitations of the present study are differences in cognitive outcome measures (clinical diagnosis vs neuroimaging vs cognitive scores), the possibility of residual confounding in non-randomized studies, and several relatively short follow-up periods in some cohorts. Also, though this review featured a large cumulative sample size, reported studies were individually much more divergent in scope and rigor. The next research steps should involve pragmatic trials with cognitive endpoints assigned as primary. A stratification according to BBB-penetrance and APOE genotypes might further clarify which ARBs are most effective in specific patients. With an aging global population and corresponding increase in dementia, the congruence of intervention in hypertension with cognitive preservation can be a significant change in preventive medicine.

## Conclusions

This comprehensive review and meta-analysis with GRADE assessment demonstrated that ARBs were associated with a modest (but significant) reduced risk of cognitive decline compared with ACEIs in hypertensive patients. The moderate-certainty evidence indicated that ARBs could be a better option in choosing the antihypertensives for those patients with increased cognitive vulnerability (e.g., metabolic syndrome or genetic predisposition).

While there was no difference between the cardiovascular benefits recorded from both drug classes, ARBs had better neuroprotective results, especially in clinical studies that used BBB crossing agents. However, the evidence was limited due to the use of observational data, and more randomized trials are needed to validate these findings in the future. In the meantime, the choice of ARBs can be considered by clinicians if the preservation of the cognitive state is a clinical goal.
